# Long-term safety and efficacy of gene-corrected autologous keratinocyte grafts for recessive dystrophic epidermolysis bullosa

**DOI:** 10.1186/s13023-022-02546-9

**Published:** 2022-10-17

**Authors:** Jodi Y. So, Jaron Nazaroff, Chinonso V. Iwummadu, Nicki Harris, Emily S. Gorell, Shivali Fulchand, Irene Bailey, Daniel McCarthy, Zurab Siprashvili, M. Peter Marinkovich, Jean Y. Tang, Albert S. Chiou

**Affiliations:** 1 Department of Dermatology, Stanford University School of Medicine, 455 Broadway Street, Room 143, MC 5338, Redwood City, CA 94063 USA; 2 Department of Dermatology, University of Cincinnati, Cincinnati, OH USA; 3 Abeona Therapeutics Inc., New York, NY USA; 4 Dermatology Division, Veterans Affairs Medical Center, Palo Alto, CA USA

**Keywords:** Epidermolysis bullosa, Recessive dystrophic epidermolysis bullosa, Genetic diseases, Genodermatoses, Blistering diseases, Gene therapy, Clinical trials

## Abstract

**Background:**

Recessive dystrophic epidermolysis bullosa (RDEB) is a rare, devastating blistering genodermatosis caused by mutations in the *COL7A1* gene, which encodes for type VII collagen and is necessary for dermal-epidermal adhesion and integrity. Disease manifestations include severe and debilitating wounds, aggressive squamous cell carcinomas, and premature death; however, there are currently no approved therapies. This Phase 1/2a, open-label study evaluated the long-term efficacy and safety of gene-corrected autologous keratinocyte grafts (EB-101) for chronic RDEB wounds.

**Methods:**

Autologous keratinocytes were harvested from participants with severe RDEB, transduced with a retrovirus containing the full-length *COL7A1* gene, and grown into 5 × 7 cm (35 cm^2^) sheets. Gene-corrected keratinocyte sheets were then transplanted onto chronic RDEB wounds present for ≥ 12 weeks.

**Results:**

Seven adult participants with severe RDEB were grafted with six sheets each (42 total sheets) onto wounds and followed for a mean of 5.9 years (range 4–8 years). Long-term improvements in wound healing and symptoms were observed. At year five, 70% (21/30) of treated sites demonstrated ≥ 50% wound healing compared to baseline by investigator global assessment. No sites with ≥ 50% wound healing were painful or pruritic, compared to 67% (6/9) of sites with < 50% wound healing (*p* < 0.001) at year five. Grafts were well-tolerated throughout long-term follow-up. No serious adverse events related to treatment were reported over a mean of 5.9 years of follow-up. No persistent systemic autoimmunity against type VII collagen or replication-competent retrovirus infections were identified, and no participants developed squamous cell carcinomas related to treatment during long-term follow-up.

**Conclusions:**

Treatment with EB-101 appears safe and efficacious, and produces long-term improvements in wound healing, pain, and itch for RDEB patients. Results from the Phase 3 randomized controlled trial are forthcoming.

***Trial registration*:**

ClinicalTrials.gov, NCT01263379. Registered December 15, 2010. https://clinicaltrials.gov/ct2/show/NCT01263379

**Supplementary Information:**

The online version contains supplementary material available at 10.1186/s13023-022-02546-9.

## Background

Recessive dystrophic epidermolysis bullosa (RDEB) is a rare, debilitating autosomal recessive disease caused by biallelic mutations in *COL7A1*, the gene encoding type VII collagen (C7) [1]. C7 is produced by basal keratinocytes and dermal fibroblasts, and is the primary component of anchoring fibrils (AF), specialized extracellular structures originating in the lamina densa that link to dermal collagen fibers to stabilize dermal-epidermal adhesion [2, 3]. In RDEB, C7 expression is typically abnormal or absent, leading to widespread subepidermal blistering and a constellation of characteristic clinical findings including extensive wounding, scarring, strictures, musculoskeletal contractures, increased risk of aggressive squamous cell carcinoma (SCC) and premature mortality [1, 4, 5]. There are currently no disease modifying treatments approved for RDEB. In recent years, several pre-clinical studies and clinical trials using gene therapy approaches aimed at correcting the underlying molecular phenotype of RDEB have been initiated [6, 7]. However, much remains unknown about the long-term outcomes and safety implications of gene therapy in RDEB including the potential risk for insertional mutagenesis and malignancy associated with retroviral vectors [8], or autoimmunity to genetically-modified cells or full-length C7 [9].

As previously reported, autologous gene-corrected keratinocytes expressing full-length C7 using a retroviral vector (EB-101, previously named LZRSE-COL7A1 Engineered Autologous Epidermal Sheets [LEAES]) were developed to treat chronic open wounds in patients with severe RDEB in the first gene therapy trial for RDEB (ClinicalTrials.gov, NCT01263379) [10, 11]. In the preliminary results of this Phase 1/2a open-label study, EB-101 was well-tolerated without any serious related adverse events during short-term follow-up. Full-length C7 expression was observed two years after grafting, demonstrating durable molecular correction of RDEB after treatment. Here, we present comprehensive, long-term (mean 5.9 years, range 4–8 years) efficacy and safety data on seven adults treated with EB-101 for severe RDEB wounds in the largest long-term follow-up study for a dermatologic gene therapy treatment to date.

## Results

Seven participants (five male and two female) with severe RDEB underwent grafting (Table 1). The mean age was 28.7 years (range 18–45) at the time of treatment. Each patient received six grafts, for a total of 42 grafts. Gene-corrected keratinocyte grafts were placed onto 38 chronic wounds that had been present for a mean of 11.2 years (range 3–20 years) and four induced wounds, as previously described [10, 11]. Participants were followed for a mean of 5.9 years (range 4–8 years).

**Table 1:** Cohort characteristics at baseline and after treatment at last follow-up

Characteristic	Subject 1	Subject 2	Subject 3	Subject 4	Subject 5	Subject 6	Subject 7
*At baseline*							
Age at baseline (years)	23	19	32	18	32	32	45
Sex	M	M	M	M	F	M	F
Grafting year	2013	2014	2014	2015	2016	2016	2017
*COL7A1* mutation 1, location	c.90delC, exon 2	c.90delC, exon 2	c.6527dupC, exon 80	c.8053 C > T, exon 109	c.4172dupC, exon 36	8440 C > T, exon 114	c.6176A > G, exon 73
*COL7A1* mutation 2, location	c.5048_5051 dup4 (GAAA), exon 54	c.5048_5051 dup4 (GAAA), exon 54	c.7485 + 5 G > A, intron 98	c.7929 + (11_26) del16, intron 106	c.4182_4188dup7, exon 36	8440 C > T, exon 114	c.6501G > A, exon 79
NC1 expression by Western blot	NC1 +	NC1 +	NC1 +	NC1 +	NC1 +	NC1 +	NC1 +
Type VII collagen expression by IF^[a]^	Undetectable NC1 and NC2	Undetectable NC1 and NC2	Trace NC1, Undetectable NC2	Undetectable NC1 and NC2	Undetectable NC1 and NC2	Undetectable NC1 and NC2	NC1 + , Undetectable NC2
Electron microscopy of skin biopsy	No mature AFs, sub-LD split	No mature AFs, sub-LD split	No mature AFs, sub-LD split	No mature AFs, sub-LD split	Rudimentary AFs, low LH24	Poorly formed AFs	Rudimentary AFs, low LH24
Circulating C7 antibodies^[b][c]^	Negative	Negative	Negative	Positive	Negative	Negative	Negative
Prior history of SCC	No	No	No	No	Yes	No	No
*After treatment at last follow-up*							
Age at last follow-up (years)	31	26	39	23	36	37	50
Follow-up period (years)	8	7	7	5	4	5	5
Presence of RCR in blood^[d]^	No	No	No	No	No	No	No
Development of SCC at any location^[e]^	No	No	No	No	Yes	Yes	No
Development of SCC at graft site	No	No	No	No	No	No	No
Vital status	Alive	Alive	Alive	Deceased	Deceased	Alive	Alive

[a] IF = immunofluorescence. All subjects were NC1 + when assayed by Western blot as required by inclusion criteria. NC1 status was assessed by IF anti-C7 LH 7.2 antibody, and NC2 status was assessed by IF with anti-C7 LH24 antibody

[b] Assayed by indirect IF using patient serum placed on monkey esophagus to detect C7 antibodies localized to dermal–epidermal junction

[c] All subjects had negative initial IIF studies for circulating C7 antibodies. In subject 4, circulating C7 antibodies were later observed in pre-treatment serum using Western blot, a more sensitive test, which was performed following evidence of immune response after treatment

[d] RCR = replication-competent retrovirus

[e] SCC = squamous cell carcinoma. SCCs were evaluated using polymerase chain reaction (PCR), and no evidence of viral vector was found in SCC samples

### Long-Term Clinical Efficacy and Patient-Reported Outcomes

**Fig. 1:**
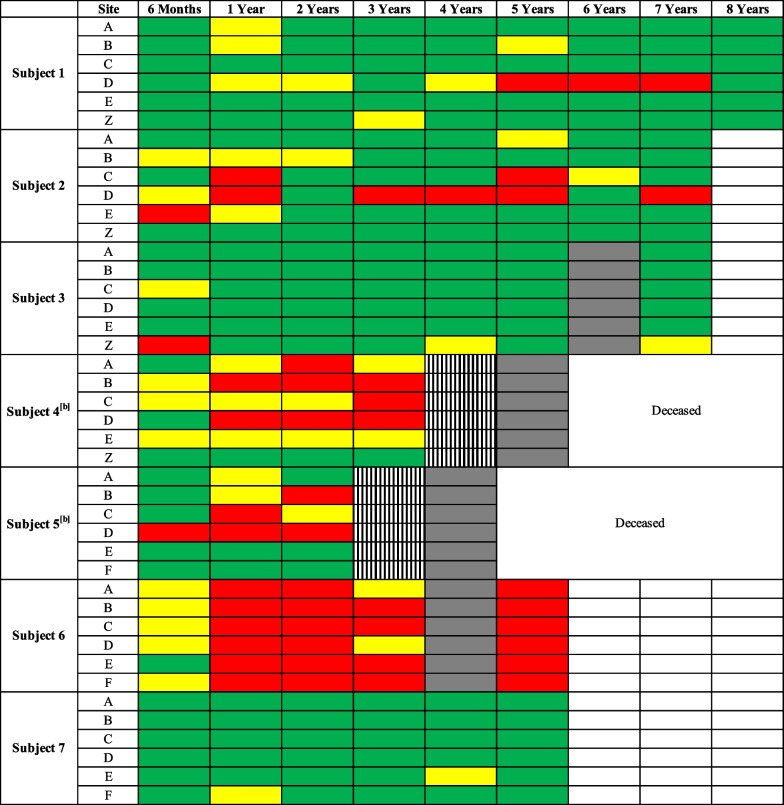
Clinical wound healing by investigator global assessment (IGA) of chronic wounds treated with gene-corrected autologous keratinocyte grafts^[a]^. [a] A red box indicates < 50% wound healing compared to baseline, a yellow box indicates 50–74% wound healing compared to baseline, and a green box indicates ≥ 75% wound healing compared to baseline. A grey box indicates that the follow-up visit was conducted virtually due to the ongoing SARS-CoV 2 pandemic, which precluded Investigator Global Assessment (IGA) of wound healing. A striped box indicates a missed appointment. A white box indicates prospective dates. Wounds A-F were chronic wounds. Wound Z was an induced wound. Control wounds are not included. [b] Subjects 4 and 5 died prior to their year six and year five visits, respectively

**Fig. 2:**
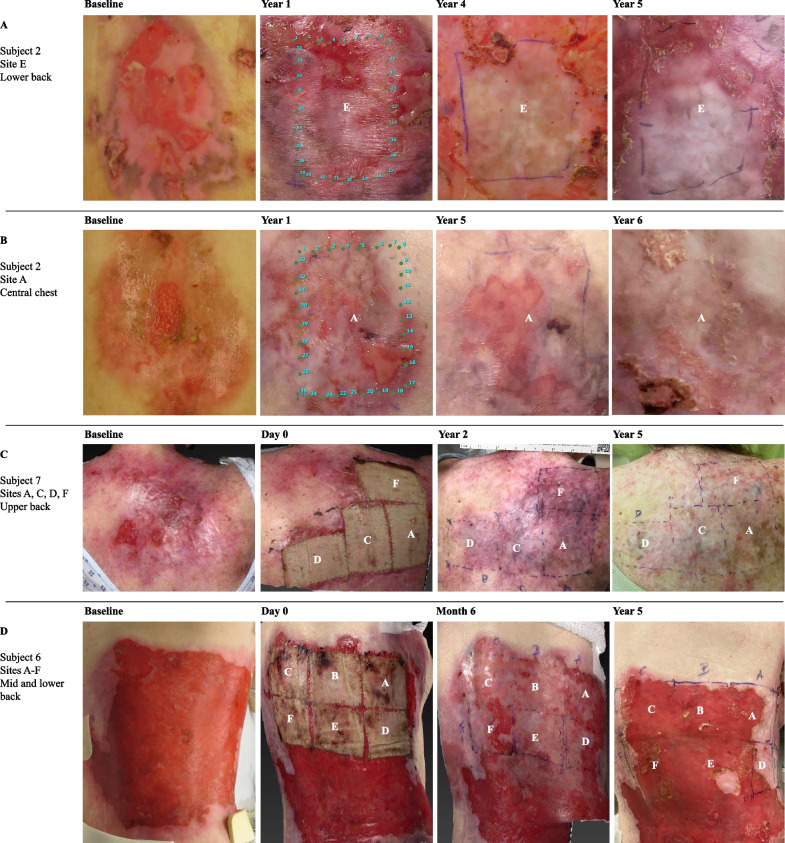
Representative photographs of chronic open RDEB wounds before and after treatment with gene-corrected autologous keratinocyte grafts. Individual 5 × 7 cm gene-corrected grafts denoted by a letter and delineated by blue dots and lines. **A** One graft was placed on a small wound on subject 2’s back. Investigator global assessment showed 50–74% wound healing compared to baseline at year one, and ≥ 75% healing at years four and five. **B** One graft applied to a small wound on subject 2’s chest demonstrated ≥ 75% healing through year four, 50–74% at year five, and ≥ 75% from year six onwards. **C** Four adjacent grafts were placed on subject 7’s upper back. ≥ 75% healing was sustained through year five. **D** Six contiguous grafts were applied to large, confluent wounds on subject 6’s back. From year one, all showed 50–74% or < 50% healing, likely due to early graft loss

Investigator global assessment (IGA) of wound healing at graft sites compared to baseline was performed at each follow-up visit, and wounds were scored as < 50% healed, 50–74% healed, or ≥ 75% healed by two independent investigators (Fig. 1) [10, 11]. Six months after application, 92.9% (39/42) of grafted sites achieved ≥ 50% wound healing. Wound healing declined somewhat during follow-up. At year five, 70.0% (21/30) of grafted sites had ≥ 50% wound healing, and 63.3% (19/30) of grafted sites had ≥ 75% wound healing. Figure 2 displays photographs of selected grafted sites at baseline and during follow-up.

**Fig. 3:**
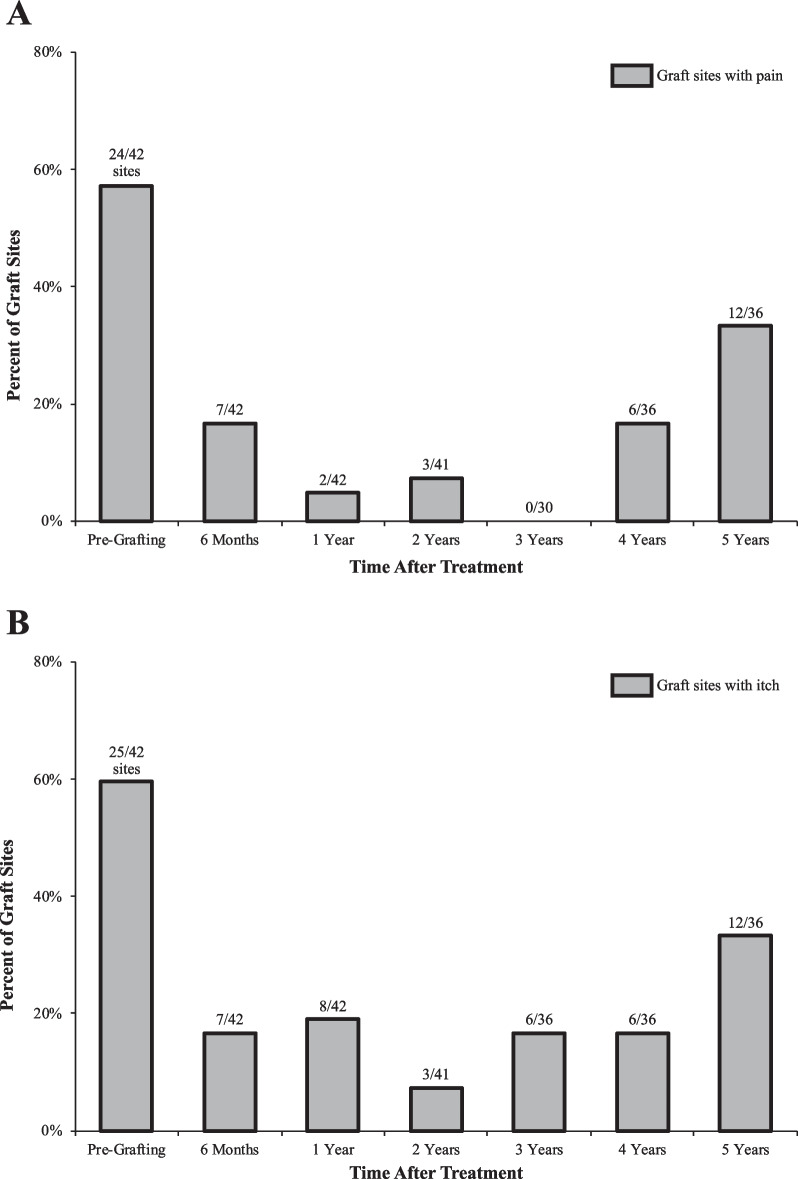
Patient-reported presence of any pain or itch at chronic RDEB wounds before and after treatment. Summary of presence of (**A**) pain (binary option: yes/no) and (**B**) itch (binary option: yes/no) at chronic, open RDEB wounds before and after treatment with 5 × 7 cm gene-corrected autologous keratinocyte grafts

Significant improvements in patient-reported outcomes (PROs) including reduced pain and itch, were sustained throughout follow-up. Figure 3 summarizes patient-reported pain and itch at grafted sites before and after treatment. At baseline, participants reported pain (binary outcome: yes/no) at 57.1% (24/42) of sites. Five years after treatment, pain was noted at 33.3% (12/36) of grafted sites. Similarly, participants reported itch (binary outcome: yes/no) at 59.5% (25/42) of sites prior to grafting, which decreased to 33.3% (12/36) of sites five years after treatment.

**Fig. 4:**
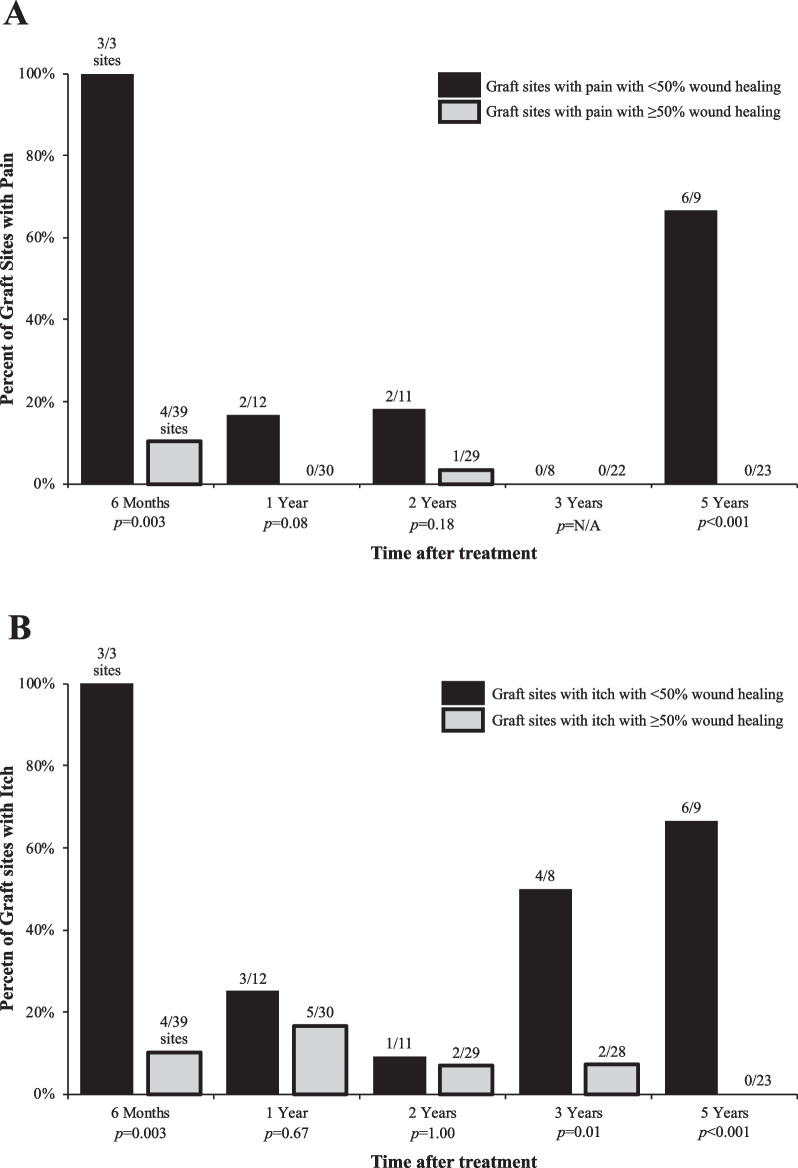
Patient-reported pain and itch at treated chronic RDEB wounds, stratified by wound healing. Summary of treated chronic RDEB wounds with (**A**) pain (binary option: yes/no) and (**B**) itch (binary option: yes/no), stratified by wounds demonstrating < 50% wound healing and ≥ 50% wound healing as determined by investigator global assessment (IGA). Statistically significant differences in pain and itch by graft site healing were estimated by Fisher’s exact test

Improved wound healing was associated with significant long-term clinical benefit in PROs. At year five, participants reported pain at 0% (0/21) of grafted sites that achieved ≥ 50% wound healing on IGA assessment compared to pain at 66.7% (6/9) of grafted sites with < 50% wound healing (Fig. 4A, *p* < 0.001). Additionally, none of the sites with ≥ 50% wound healing at year five were pruritic, however 66.7% (6/9) of sites with < 50% wound healing at year five were pruritic (Fig. 4B, *p* < 0.001). At year five, participants also reported improved skin durability compared to baseline at 52.8% (19/36) of sites and reduced blistering following trauma at 48.6% (17/35) of sites. No treated sites were less durable or had more blistering following trauma.

Beginning in August 2020, participants completed annual questionnaires which surveyed changes in pain at grafted sites compared to baseline on a seven-point Likert-type scale. At last follow-up, these graded pain surveys revealed significant reductions in pain at grafted sites compared to control sites. Compared to baseline, pain was “very much improved” or “much improved” at 47.2% (17/36) of grafted sites, and “a little improved” at 16.7% (6/36) of grafted sites (*p* = 0.003). No treated wounds had “worse” pain compared to baseline. In contrast, no control wounds had improvements in pain; 66.7% (4/6) of control wounds had “no change” in pain, and 33.3% (2/6) of control wounds had “worse” pain than baseline.

### Long-term safety

Five participants experienced serious adverse events during the long-term follow-up period until April 2022, all of which were assessed by investigators as not related to treatment. Two participants (subjects 4 and 5) died during follow-up due to RDEB disease progression. Subject 4 died from sepsis, and subject 5 died from failure to thrive due to severe malnutrition.

Two participants (subjects 5 and 6) developed cutaneous SCCs which were deemed unrelated to grafting as all occurred on anatomic locations distant from grafted sites. Subject 5, who had a known history of SCCs prior to grafting, developed nine SCCs on the bilateral upper and lower extremities on non-grafted sites during the study period. A lung nodule consistent with possible metastasis was also identified on CT scan, though a biopsy of this lesion was never performed per subject 5’s preference. Subject 6 developed eight non-graft-related, recurrent SCCs on the right arm, back, and flank, and one metastasis to an axillary lymph node [12] during follow-up. Two tumor samples (one from a primary tumor on the forearm from subject 5 during year one, and one from an in-transit metastasis on the arm from subject 6 during year two) were available for analysis via polymerase chain reaction (PCR) for the presence of replication-competent retrovirus (RCR). Both samples were negative for RCR. No other cutaneous or extracutaneous malignancies were identified to date.

Twenty-one treatment-related adverse events were reported (Table 2). The majority (85.7%, 18/21) were assessed as mild. All adverse events either self-resolved or resolved with treatment. The most common adverse events were presence of tissue-bound antibodies at graft sites (38.1%, 8/21), immediate postoperative pain at graft sites (19.0%, 4/21), pruritus (14.3%, 3/21), and immediate postoperative infection at graft sites (14.3%, 3/21).

**Table 2:** Summary of treatment-emergent adverse events related to EB-101^[a]^

		Related to Treatment			Severity Grade					Outcome	
	Total, No. (%)	Definitely, No. (%)	Probably, No. (%)	Possibly, No. (%)	Grade 1: Mild, No. (%)	Grade 2: Moderate, No. (%)	Grade 3: Severe, No. (%)	Grade 4: Life-Threatening, No. (%)	Grade 5: Death, No. (%)	Resolved Without Treatment, No. (%)	Resolved With Treatment, (No. (%)
	21	4 (19.0)	12 (57.1)	5 (23.8)	18 (85.7)	2 (9.5)	1 (4.8)	0 (0.0)	0 (0.0)	12 (57.1)	9 (42.9)
Immediate postoperative skin pain at graft site	4 (19.0)	–	1 (8.3)	3 (60.0)	3 (16.7)	–	1 (100.0)	–	–	–	4 (44.4)
Pruritus	3 (14.3)	–	3 (25.0)	–	3 (16.7)	–	–	–	–	3 (25.0)	–
Immediate postoperative graft site infection within 10 days of treatment	3 (14.3)	–	2 (16.7)	1 (20.0)	2 (11.1)	1 (50.0)	–	–	–	2 (16.7)	1 (11.1)
Postoperative graft site infection 6 months after treatment	1 (4.8)	–	–	1 (20.0)	–	1 (50.0)	–	–	–	–	1 (11.1)
Presence of tissue-bound anti-C7 antibodies at grafts	8 (38.1)	3 (75.0)	5 (41.7)	–	8 (44.4)	–	–	–	–	5 (41.7)	3 (33.3)
Presence of circulating antibodies in serum	2 (9.5)	1 (25.0)	1 (8.3)	–	2 (11.1)	–	–	–	–	2 (16.7)	–

[a] Includes adverse events considered "definitely", "probably", and "possibly" related to grafting that occurred after the date of treatment. Adverse events characterized using the National Cancer Institute’s Common Terminology Criteria for Adverse Events version 4.0

Two participants developed localized infections at graft sites subsequent to the immediate postoperative period at months six and twelve. Both infections resolved with oral antibiotics and did not appear to impede long-term wound healing.

Circulating anti-C7 antibodies were observed in two participants, which resolved without intervention by year one as previously reported [10, 11]. These patients did not exhibit new fevers, worsening of generalized blistering outside of grafted areas, anaphylaxis, elevated liver enzymes, or abnormal kidney function, which would be concerning for a potential systemic immune response throughout long-term follow-up.

Serum samples were analyzed at months 1, 3, 6, and annually thereafter through year five for the presence of RCR. No evidence of systemic RCR infection was ever identified in 41 samples. Systemic autoimmunity, as defined by the serologic presence of anti-C7 cytotoxic T cells, was not seen in 21 serial assays.

As previously reported, persistent tissue-bound immunoreactants at graft sites were identified in one participant (subject 4) through year two, and a subsequent Western blot analysis of this subject’s pre-treatment serum revealed the presence of anti-C7 antibodies at baseline, despite an initial negative result of baseline indirect immunofluorescence (IIF) [10, 11]. Reassuringly, this participant did not display clinical symptoms during five years of follow-up concerning for systemic autoimmunity.

## Discussion

We previously presented safety and wound outcomes for a mean of 2.7 years of follow-up in seven adult subjects with severe RDEB treated with autologous gene-corrected keratinocyte grafts [10, 11]. We now report clinical safety and efficacy data from a mean of 5.9 years (range 4–8 years) of follow-up in this Phase 1/2a trial. Our findings demonstrate that EB-101 is a safe, effective, and long-term treatment for chronic RDEB wounds.

During up to eight years of follow-up, no serious adverse events related to treatment were observed. Concerns for oncogenesis have previously been raised regarding the use of retroviral vectors for gene therapy following early clinical trials for X-linked severe combined immunodeficiency [8]. Reassuringly, no participants developed cutaneous or extracutaneous malignancies related to gene therapy and no systemic RCR infections were identified on serial serologic assays during follow-up. Two subjects developed cutaneous SCCs. However, all tumors were distant from graft sites, contained no detectable retroviral genome using PCR analysis when sampled, and arose when both subjects were in their 30 s. This strongly suggests that these malignancies developed due the subjects’ underlying RDEB, given the well-known increased risk of aggressive SCCs in adults with RDEB [13], and not due to retroviral insertional mutagenesis or oncogenesis associated with EB-101 treatment.

The long-term risk for clinically significant autoimmunity to gene-corrected keratinocyte grafts was also low. No cytotoxic T cells with anti-C7 activity were identified on serial serologic analysis. Transient circulating anti-C7 antibodies were detected in two participants. Tissue-bound antibodies beyond trace staining were detected in four participants, the majority of which were transient or resolved by year one, and no subjects developed any concerning clinical symptoms related to these localized immune reactions. In subject 4, localized immunoreactants were observed at graft sites up to two years after treatment. As previously discussed [10, 11], this participant was initially negative for pre-existing anti-C7 antibodies during enrollment screening with an immunofluorescence microscopy assay certified by the Clinical Laboratory Improvement Amendments, but was later found to have preexisting anti-C7 antibodies at baseline using a more sensitive Western blot assay. Subject 4 died five years after treatment; however, circulating anti-C7 antibodies were not thought to contribute to his death, as this participant never developed any clinical symptoms or sequelae concerning for a severe systemic immune reaction including fevers, increased generalized blistering outside of his graft sites, or anaphylaxis, and did not require any treatment for this immune response throughout five years of follow-up. Given this experience, however, enrollment criteria for the ongoing Phase 3 randomized controlled trial (ClinicalTrials.gov, NCT04227106) was revised to exclude participants with pre-existing anti-C7 antibodies at baseline as detected by either IIF or the more sensitive method of enzyme-linked immunosorbent assay (ELISA).

Wound healing varied between participants. In serial evaluations of 42 total grafted wounds, we observed that several wound and participant characteristics may affect graft uptake and contribute to poorer wound healing over time, such as the presence of persistent, localized anti-C7 antibodies. In subject 4, persistent expression of tissue-bound anti-C7 antibodies at graft sites may have led to early and ongoing degradation of full-length C7 expressed at grafts, resulting in reduced long-term molecular correction. However, the transient expression of localized antibodies in subjects 1, 2, and 3 did not appear to impede long-term wound healing.

Larger baseline wound size and anatomic location also impacted wound healing. In subject 6, all six grafts were placed contiguously on a large (> 200 cm^2^), confluent wound bed on the mid and lower back (Fig. 2D). Starting at year one, subject 6 had < 50% wound healing at most of their graft sites (Fig. 1), which likely represents poor graft uptake and early graft loss due to mechanical trauma to the grafts as this location was more difficult to immobilize and protect from excess friction and pressure during the immediate postoperative period compared to other anatomic locations, such as the extremities. Given the poor graft uptake observed in subject 6, the postoperative care protocol was revised for the ongoing Phase 3 randomized controlled trial (ClinicalTrials.gov, NCT04227106) to optimize graft uptake, including standardizing the required postoperative inpatient admission period to at least one week with strict immobilization of graft sites and extensive graft bandaging, padding, and care by research staff to reduce trauma, pressure, and friction at graft sites.

However, other grafts placed on areas at risk for trauma or friction successfully demonstrated long-term wound healing. Subject 7 received four contiguous grafts on the upper back and posterior shoulder (Fig. 2C), and by year 5, sustained ≥ 75% wound closure at all treated sites (Fig. 1). The success of subject 7’s grafts may be due to the characteristics of her open wounds at baseline which, in contrast to subject 6’s wounds, were discrete (i.e., not confluent) and smaller, with an average open wound size of 25 cm^2^. Similarly, subject 2 received a graft on a single wound on the central low back (Fig. 2A) which demonstrated ≥ 75% wound healing from years two onwards; the success of this graft site may also be attributed to its smaller baseline wound area. The long-term wound healing observed on graft sites on the back for subjects 2 and 7 suggests that successful, durable re-epithelialization of areas at risk for trauma and ulceration is possible, though may be influenced by wound-specific factors including smaller baseline wound size. These findings are consistent with prior work which identified that larger RDEB wounds are more difficult to close compared to smaller wounds [14, 15], and highlight the need for careful consideration of wound characteristics including location and baseline size when selecting wounds for clinical trials.

Wound healing at individual graft sites also varied over time. Subject 2’s wound E, for example, showed < 50% wound healing at month six, but improved to ≥ 50% healing at year one, and demonstrated ≥ 75% healing from year two onwards as noted above. Mechanisms underlying these fluctuations in wound healing within a single graft site are multifactorial, and may include recent trauma, and bacterial colonization or infections [16]. Nonetheless, the observed, new capacity for these previously chronic open wounds to repeatedly heal after grafting demonstrates that treatment with EB-101 may improve long-term skin durability and wound healing.

Notably, increased wound healing was significantly correlated with sustained reductions in pain and itch. Wounds with improved wound healing were significantly less painful and less pruritic than wounds with poorer wound healing, demonstrating that treatment with gene-corrected keratinocyte grafts is associated with long-term, clinically-significant benefit for patients with RDEB, even in the absence of complete wound closure. As much remains unknown about the natural history and progression of wounds in RDEB [15, 17], the use of PROs including pain and itch in therapeutics targeting chronic wounds allows investigators to incorporate the participant’s own perspective and experiences of investigational treatments [18], and to target drug development towards treatments that are clinically meaningful for patients.

These findings have several implications for clinical care, as chronic wounds are a major mediator of disease course in RDEB. The disrupted microenvironment and pathologic remodeling of chronic wounds leads to persistent inflammation and increased risk of bacterial colonization and infection, facilitating the development of sepsis, as well as severe, treatment-refractory anemia, and malnutrition due to increased metabolic demand from impaired wound healing [16, 19]. This, in turn, can worsen disease trajectory by further impairing wound healing capabilities and physiologic reserve. Critically, aggressive SCCs, which are the leading cause of death in adult RDEB patients, frequently develop at chronic wounds [13]. Chronic wounds are also associated with poorer psychosocial outcomes [20] as chronic wounds are significantly larger and more painful than recurrent wounds [15]. Chronic wounds are also associated with worse quality of life [21]. Lastly, chronic wounds impose significant financial and time burdens on RDEB patients due to the need for extensive routine wound dressing changes [19, 22]. Thus, treatment of chronic RDEB wounds may produce many important clinical benefits, including improvements in both disease course and outcomes, and quality of life.

A case report of transgenic epidermal grafting for junctional epidermolysis bullosa (JEB) recently reported long-term outcomes for one patient with JEB who received grafts expressing full-length, corrected *LAMB3*, which encodes for laminin-332 [23, 24]. Large, full-body grafts were placed on extensive wounds, and remained intact after five years. As detailed previously [6], laminin-332 promotes keratinocyte stem cell maintenance and growth [25], and directly mediates keratinocyte adhesion [26]; C7, notably, lacks these qualities, which may have contributed to the more variable durability of *COL7A1*-corrected grafts observed for RDEB in the present study. Future study of gene therapies for RDEB must consider these intrinsic characteristics of RDEB biology, including investigating methods to optimize transduction and enrichment of keratinocyte stem cells within grafts to improve long-term graft durability.

Our findings are limited by the small sample size, a common challenge in clinical trials for rare or orphan diseases, such as RDEB. This Phase 1/2a clinical trial focused on long-term safety and clinical efficacy; thus, serial skin biopsies to evaluate molecular expression of full-length C7 within grafts was not performed beyond year two [11], which limited definitive assessment of long-term molecular correction of C7. Nonetheless, the observed, sustained wound healing and reduction in pain and itch years after grafting suggests that gene-corrected keratinocyte grafts may confer significant long-term benefits, and further evaluation of the specific molecular etiology of these benefits may be warranted. To reduce the risk of autoimmunity and graft rejection, all subjects in this trial were also required to demonstrate sufficient expression of the NC1 domain [10, 11, 27]. This limits the generalizability of these results, particularly among patients with null *COL7A1* mutations who lack any C7 expression. Another limitation was the categorization of wound healing by IGA rather than quantification of wound healing using photography software as this was the outcome measure recommended by the FDA. Some observed fluctuations in wound healing at a single wound site over time may be attributed to the categorical nature of this scoring methodology; for example, Subject 5’s graft A was scored by IGA as ≥ 75% wound healing at month six, and 50–74% wound healing at year one. When wound healing at this site was evaluated on a continuous scale using the Canfield Vectra 3D photography system (Canfield Scientific, Parsippany, NJ), however, it was assessed as 96% wound healing at month six, and 70% wound healing at year one—a value which is close to the 75% wound healing cutoff used in the categorical IGA methodology. Lastly, this trial was initially designed with a focus on safety outcomes and a limited number of control wounds were selected. Thus, wound pairs were not randomized prior to treatment, and systematic comparisons of control and treated wounds could not be performed.

## Conclusions

In conclusion, autologous gene-corrected keratinocyte grafts may be a safe, durable treatment for chronic RDEB wounds, and the results of this Phase 1/2a trial demonstrate early evidence of sustained, long-term clinical benefit for patients with RDEB. These results provide support that ex vivo cell therapy strategies may be viable approaches for RDEB and other monogenic genodermatoses. The Phase 3 randomized controlled trial (ClinicalTrials.gov, NCT04227106) began in January 2020 and recently completed enrollment. Results are forthcoming.

## Methods

### Study design and approval

This study was a Phase 1/2a, single-center, non-randomized, open-label clinical trial investigating autologous, gene-corrected keratinocyte sheets expressing full-length C7 to treat chronic wounds in RDEB. Details regarding study design including inclusion/exclusion criteria and procedures have been described previously [10, 11]. The study methodology is described here in brief. This study was approved by the Stanford University Institutional Review Board and the Food and Drug Administration.

### Study ethics, inclusion criteria, and exclusion criteria

Written informed consent was obtained from all study participants prior to enrollment. Adults with RDEB who had two confirmed *COL7A1* mutations (GeneDx, Gaithersburg, MD) were selected for screening as previously described [27]. Detectable expression of the NC1 domain of C7 was required for study inclusion to reduce the risk of immunologic response to full-length C7 expressed within the epidermal grafts, as the NC1 domain is considered the most immunogenic portion of the C7 protein [27, 28]. Exclusion criteria included pre-existing anti-C7 antibodies, prior or current SCC at sites selected for grafting, and significant medical complications or illnesses. Seven patients ultimately received treatment (Additional file 1: Fig. S1).

### Treatment

Autologous keratinocyte grafts were developed from skin biopsies and transduced with a retroviral vector carrying full-length *COL7A1*, as previously discussed [10, 11]. Chronic wounds present for ≥ 12 weeks and with a total open area of 100 cm^2^ or larger were identified for treatment. Six grafts per participant were surgically placed onto treatment sites under general anesthesia. Treatment sites were selected based on subject preference, ease of surgical access, and ease of postoperative care and immobilization. Non-randomized chronic wounds were selected as control wounds and received standard wound care.

### Follow-up

Participants were monitored at months 3, 6, 9, and 12 following treatment and once a year thereafter. Due to travel restrictions from the ongoing SARS-CoV-2 pandemic, some follow-up visits were conducted as virtual visits. Wound healing compared to baseline was assessed by IGA of graft sites by two independent dermatologists (wound scoring: < 50% healed, 50–74% healing, or ≥ 75% healed) and photographs were obtained. PROs were captured including the presence of pain (options: yes/no) and pruritus (options: yes/no) of grafted and control sites, current wound durability compared to baseline (options: more durable, the same, or less durable), and ease of blistering after trauma compared to baseline (options: more difficult to blister, no change in ease of blistering, or easier to blister). An additional survey was subsequently developed and administered to participants annually beginning in August 2020 to capture graded changes in pain following treatment using a Likert-type scale (options: very much improved, much improved, minimally improved, no change, minimally worse, much worse, very much worse, or do not remember).

Safety outcomes including systemic or local immune response, infection, development of SCC, and presence of RCR were assessed annually as previously described [10, 11]. Systemic immune responses were assessed via immunofluorescence for serum-bound immunoreactants (IgA, IgG, IgM, and C3), and serum assays for cytotoxic T cells against C7 were performed. Participant serum was also tested for the presence of RCR DNA via PCR with retrovirus-specific primers in accordance with guidelines from the Food and Drug Administration [29]. Adverse events were assessed and graded using the National Cancer Institute’s Common Terminology Criteria for Adverse Events version 4.0 (https://ctep.cancer.gov/protocolDevelopment/electronic_applications/ctc.htm).

Samples of new SCCs were obtained for analysis where possible to evaluate for the presence of RCR. SCC biopsy specimens were evaluated with PCR using multiple primer sets specific for the proviral genome extended packaging sequence (5’ primers) and sequence within exon 2 of *COL7A1* cDNA (3’ primers).

### Statistical analysis

Counts and percentages were calculated based on the number of wounds assessed at each time point. Fisher’s exact test was performed to estimate differences in PROs. All *p*-values were two-sided, and *p* < 0.05 was considered statistically significant. Statistical analyses were performed using StataSE 16.1 (College Station, TX).

## Supplementary Information

**Additional file 1.  Figure S1**: Study enrollment diagram. Legend: CONSORT diagram of participant enrollment for this Phase 1/2a clinical trial.

## Data Availability

Data sharing is not applicable to this article as no datasets were generated or analyzed during the current study.

## Abbreviations

RDEB: Recessive dystrophic epidermolysis bullosa
C7: Type VII collagen
AF: Anchoring fibril
SCC: Squamous cell carcinoma
LEAES: LZRSE-COL7A1 Engineered Autologous Epidermal Sheets
IGA: Investigator global assessment
PRO: Patient-reported outcome
PCR: Polymerase chain reaction
RCR: Replication-competent retrovirus
ELISA: Enzyme-linked immunosorbent assay
JEB: Junctional epidermolysis bullosa
