# Supplementary figures and images for: Long-term safety and efficacy of gene-corrected autologous keratinocyte grafts for recessive dystrophic epidermolysis bullosa
Source: Orphanet J Rare Dis. 2022 Oct 17;17:377. doi: 10.1186/s13023-022-02546-9 (PMC9574807; doi:10.1186/s13023-022-02546-9)

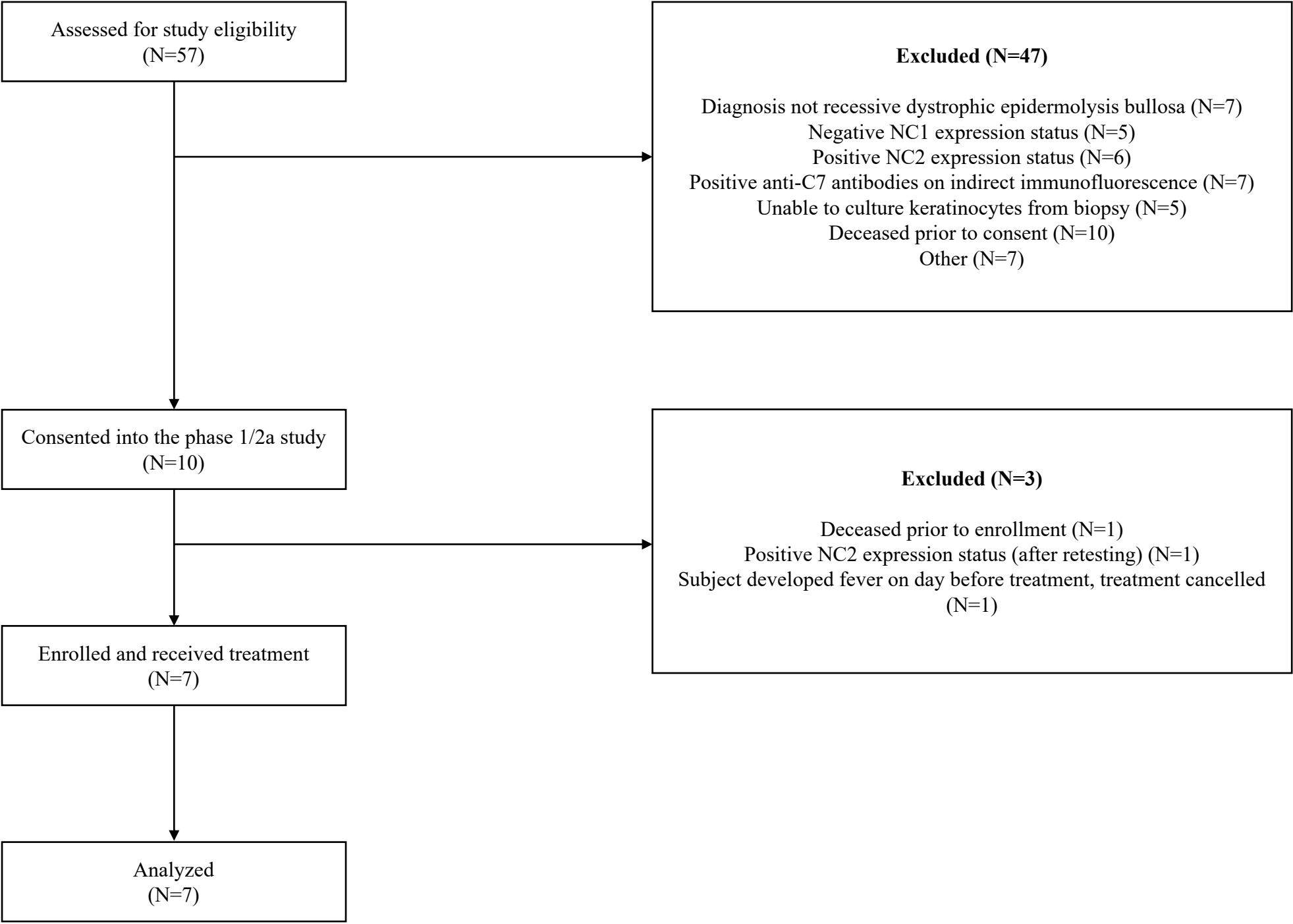

Supplement: Supplementary file 1 — Additional file 1. Figure S1: Study enrollment diagram. Legend: CONSORT diagram of participant enrollment for this Phase 1/2a clinical trial. [file 13023_2022_2546_MOESM1_ESM.pdf]
